# Unraveling LncRNAs: the future of lung cancer treatment

**DOI:** 10.1186/s43046-025-00327-6

**Published:** 2025-10-26

**Authors:** Zahraa Isam Jameel, Halla Abdul-Hadi Chabuk

**Affiliations:** 1 Department of Biology, College of Science, Al-Qasim Green University, Al‑Qasim, Babil Iraq; 2 Department of Biology, College of Science, University of Babylon, Babil, Iraq

**Keywords:** LncRNAs, Lung cancer, Metastasis, Biomarker, Dysregulation

## Abstract

Long non-coding ribonucleic acids (LncRNAs) are larger than 200 nucleotides and resemble messenger ribonucleic acids (mRNAs), but they do not code for proteins. In both cell development and physiological cell function, LncRNAs have crucial biological functions. Consequently, cancer entails the disruption of their biological function. Many people die from lung cancer because it is diagnosed late, spreads to other parts of the body, and has a high treatment failure rate. Because they can be involved in either oncogenic or tumor-suppressing functions, LncRNAs are quickly becoming core molecules in lung cancer. Since LncRNAs are long-lasting in blood, they can be utilized as non-invasive diagnostic tools for cancer at an early stage. We review the latest research that has brought together evidence from real-world observations concerning the processes through which LncRNAs work in cancer formation, how they allow cancer to develop drug resistance, and how they can be used as possible diagnostic tools and markers of outcome, with a focus on lung cancer. We also cover some of the ongoing treatment strategies that can target LncRNAs. As seen from what has been laid out here, the examination of LncRNAs in lung cancer with protein-coding genes could provide evidence for a further elucidation of the molecular events behind the disease as well as its progression, and the potential for a new therapeutic pathway.

## Introduction

About one-fifth of all cancer fatalities occur from lung cancer, making it the top cause of cancer-related mortality globally [1, 2]. Between 85 and 90% of lung cancer cases are non-small cell lung cancer (NSCLC) [3]. The absence of reliable early diagnostic indicators and the emergence of resistance to targeted therapies or chemotherapy in later stages of the disease are the main causes of the low survival rates experienced by patients with lung cancer [4, 5]. New insights from molecular biology have shown that LncRNAs play an important part in regulating gene expression and have important implications for cancer biology, particularly lung cancer.

The general definition of RNAs that are longer than 200 nucleotides but do not often code for proteins is long non-coding RNAs, or LncRNAs. Polymerase II transcribes these 5-capped molecules, which resemble messenger RNA (mRNA) and have a 3-poly-A tail. They serve a purpose because of the intricate secondary structures they are capable of forming [6–8]. Many long non-coding RNAs are found in the nucleus and help manage the structure of chromatin and the process of transcription, often by forming lncRNA-DNA triplexes, nuclear speckles, and assisting in splicing. Long non-coding RNAs (lncRNAs) have several functions in the cytoplasm, including controlling the stability of messenger RNAs (mRNAs), binding to other non-coding RNAs, and influencing the post-translational modifications and actions of proteins [9–13].

Short non-coding RNAs (ncRNAs) and long non-coding RNAs (LncRNAs) are classified according to the length of the RNAs in terms of nucleotides. Long non-coding RNAs (LncRNAs) are among the most diverse and abundant classes of RNAs; their average length is more than 200 nucleotides. Most of the non-coding RNAs are LncRNAs, but they cannot be utilized for coding proteins [14–16]. Lung cancer is one of many disorders implicated in LncRNA dysregulation, loss, or mutation, according to recent research. Long non-coding RNAs (LncRNAs) control gene expression by their interaction with microRNAs, LncRNAs with protein, mRNA splicing, epigenetic alteration, and LncRNAs with other LncRNAs. Intracellular localization of LncRNA also determines mechanisms through which they modulate tumor cell growth. Through their role as structural frameworks to hold nuclear domains and chromatin structure and transcription regulators, nuclear LncRNAs regulate biological processes and modulate biological functions [17]. In contrast, cytoplasmic LncRNAs regulate post-transcriptional modification and messenger RNA translation, impacting cell signaling [18]. The most prevalent type of lung cancer, non-small cell lung cancer (NSCLC), is the leading cancer-related mortality globally. Advanced lung cancer patients are more likely to have distant metastases at the time of discovery, and only 9% of such patients live 5 years [19].

Recent developments in target therapy (tyrosine kinase inhibitors, TKIs) have been advantageous to patients, but natural and acquired resistance has a tendency to limit the efficacy of these therapies, leading to tumor development and recurrence, hence poor survival rates.

In order to set up better therapeutic strategies, knowledge regarding the molecular mechanism of lung cancer carcinogenesis, metastasis, and drug resistance should be known. Their tumor-suppressive or oncogenic activities are also rendering them star cancer molecules. In this article, here we review the most current findings that have examined lncRNAs in lung cancer, drug resistance, and their prospective biomarker roles in vivo.

## LncRNAs and the hallmark of lung cancer

**Fig. 1:**
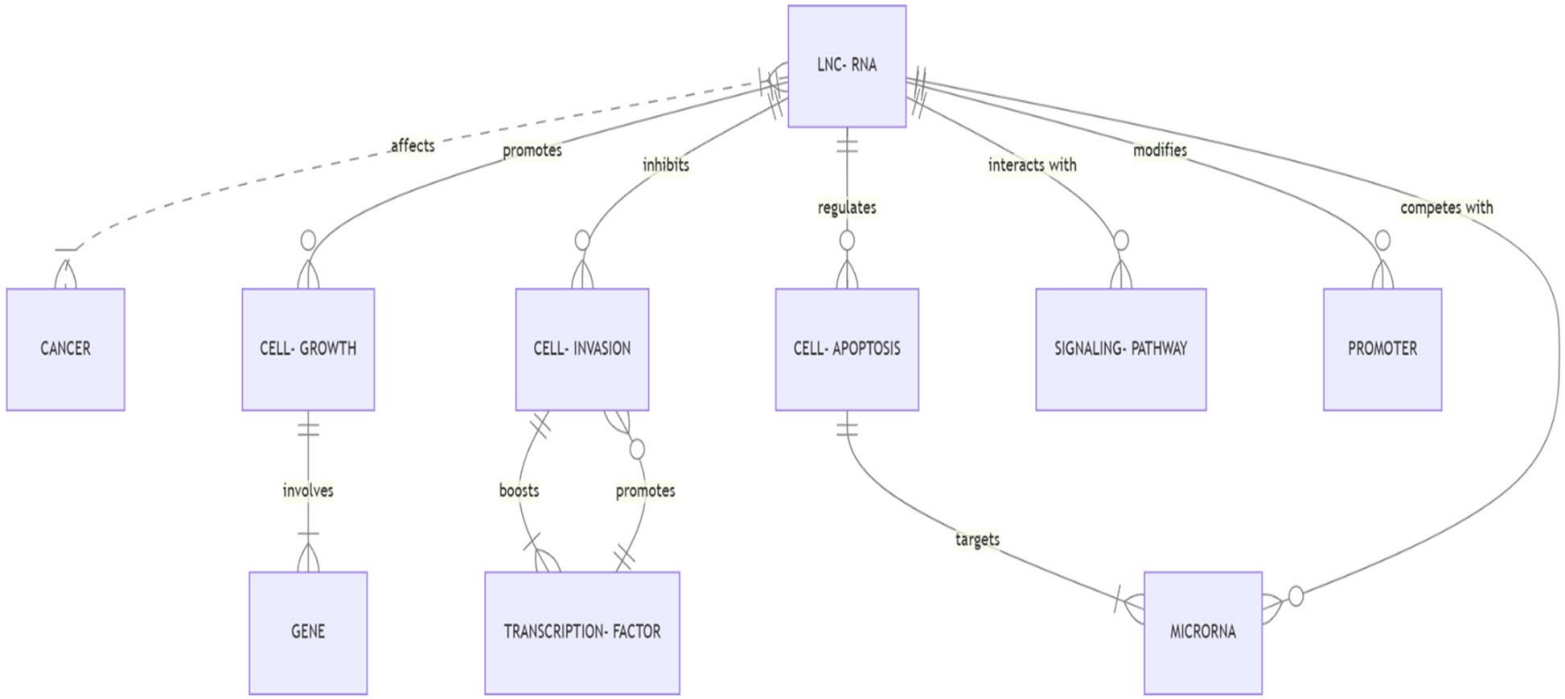
Functions of lncRNAs. Numerous biological processes are impacted by the crucial functions of long non-coding RNAs. In the nucleus, long non-coding RNAs have a role in controlling splicing, transcription, and epigenetic mechanisms. On the flip side, long non-coding RNAs (lncRNAs) in the cytoplasmic domain regulate mRNA translation, serve as modulators for tiny regulatory RNAs, and may even produce small peptides. Other functions include Scaffolding: LncRNAs can act as scaffolds, bringing multiple proteins together to form functional complexes. Decoys: LncRNAs can bind to proteins or other RNAs, preventing them from interacting with their target ligands. Guides: LncRNAs can guide proteins to target DNA or RNA locations, regulating gene expression or RNA processing. Signals: LncRNAs can act as signals, reporting the existence of specific cellular states or stimuli

Cell proliferation, metastasis, apoptosis, and angiogenesis are some of the hallmarks of non-small cell lung cancer (NSCLC), all of which rely on LncRNAs. To understand the progression of lung cancer and to create targeted treatments, it is essential to understand the regulatory functions they play in these processes. Many of the ways that control oncogenes and tumor suppressors, like DNA methylation, gene amplification or deletion, mutations, and LncRNA expression and function, are also linked to cancer. New biochemical and bioinformatics techniques have also shown that LncRNAs that were not thought to code for anything may in fact encode tiny peptides with biological activity. These coding products greatly influence the development of NSCLC. This section will discuss the LncRNAs depicted in Fig. 1, which either play an oncogenic or tumor-suppressing role in NSCLC.

### Cell proliferation and cell cycle regulation

Abrogation of apoptotic signaling pathways and cell cycle is usually involved in the initiation and progression of malignant tumors. Newly emerging evidence suggests potential functions of LncRNAs in the initiation and progression of tumors, as initiators or inhibitors. LncRNAs, similar to protein-coding genes, can be divided into subgroups that either facilitate or inhibit tumor progression. A compilation of molecular actions through which LncRNAs act to influence cancer growth is given below. They include their roles as coding peptides, protein scaffolds, and miRNA sponges. Overexpression of tumor-like biological activities is induced by LncRNA-XIST. By competitive binding with miR-16 in the ceRNA network, XIST supersedes repression of the target gene CDK8 downstream, as indicated by Sabnis and Bivona. This interaction promotes tumor proliferation and growth by keeping the cells in the G0–G1 phase of the cell cycle [20]. Enrichment of XIST and miR-186-5p in immunoprecipitation of Ago2 was also supported in RIP studies. Through the modulation of the cell cycle, XIST inhibits the tumor-suppressive action of miR-186-5p and influences proliferation of cells [21]. In NSCLC cell lines, lncRNA-HOTAIR is overexpressed, according to Chen et al. Downregulation of HOTAIR increases the expression of miR-217, which again inhibits the proliferation of A549 [22]. In addition, HOTAIR promotes the proliferation and invasion of NSCLC cells by acting as a competitive endogenous RNA for miR-149-5p in most researches [23].

The majority of tumor tissues show elevated expression of MALAT1, one of the first dysregulated lncRNAs in NSCLC to be discovered. MALAT1 increases cell proliferation by regulating gene expression associated with the cell cycle, as summarized in Table 1. It speeds up cell division by interacting with a number of transcription and splicing factors that trigger the G1-to-S phase transition. In multiple NSCLC cell lines, Li et al. [23] found that MALAT1 was markedly overexpressed. Reducing MALAT1 expression in A549 and H460 cell lines led to a rise in miR-124 levels, which in turn suppressed MALAT1 expression. Ago2 was found to be substantially enriched for miR-124 and MALAT1, suggesting that these two interact with one another, according to the experimental data. So, NSCLC cell proliferation is enhanced by the MALAT1/miR-124/STAT3 pathway [24]. A549 and H460 cell lines showed significant expression of MALAT1 and MDM4, and there was a positive connection in NSCLC tissues, according to another study [25]. By decreasing miR-185-5p levels, MALAT1 promotes NSCLC cell proliferation by increasing the expression of MDM4, its target gene [26]. Overexpression of DANCR in NSCLC increases EZH2 binding to p21, which in turn increases H3K27me3 alteration at the p21 promoter region, suppresses p21 expression, and facilitates NSCLC cell proliferation [27]. According to Yu et al., DANCR promotes cell proliferation via the Wnt/β-catenin signaling pathway by competing with miR-216a to regulate β-catenin expression [28]. Based on these findings, Meng et al. concluded that JPX controls cell cycle progression by interacting with miR-145-5p and upregulating downstream cyclin D2 production via the ceRNA pathway [29]. Furthermore, SNHG15 blocks miR-486 from binding, which increases CDK14 mRNA expression and impacts the proliferation and development of NSCLC cells [30]. One mechanism by which XIST prevents cell death is via binding to miR-449a, which increases levels of the anti-apoptotic protein Bcl-2 [31, 32].

**Table 1:** Multiple LncRNAs (MALAT1, HOTAIR, DANCR) and role in lung cancer

LncRNAs type	Function
MALAT1 (Metastasis-Associated Lung Adenocarcinoma Transcript 1)	- Regulation of splicing alternative: MALAT1 binds to splicing factors, which in turn regulate alternative splicing of pre-mRNAs and produce protein isoforms that promote survival and growth of cancer cells - Cell proliferation and migration pathways are induced to lead to metastasis and tumor development, something that MALAT1 is capable of. MALAT1’s interaction with chromatin-modifying enzymes enables it to control gene expression in reaction to cell cycle alterations and cell death
HOTAIR (HOX Transcript Antisense Intergenic RNA)	- Recruitment of the Polycomb Repressive Complex 2 (PRC2): HOTAIR is also bound by this complex. PRC2 is a group of histone methyltransferases that silence gene expression - The HOTAIR pathway can promote carcinogenesis by inhibiting the expression of tumor suppressor genes through recruitment of PRC2 to target genomic sites - The epithelial cells can also become less adhesive to one another and more migratory and invasive because of the ability of HOTAIR to induce epithelial-mesenchymal transition (EMT). EMT is an important process in cancer dissemination
DANCR (Differentiation antagonizing non-protein coding RNA)	- Inhibiting cell differentiation: DANCR can inhibit cancer cells from differentiating into another type of cells and instead continue to proliferate and grow continuously - One of the ways that DANCR controls the function of miRNA is by binding to miRNAs and inhibiting their binding to their target mRNAs. Downregulation of tumor suppressors and upregulation of oncogenes can be the result of this

### Metastasis and invasion

Major determinants of the poor prognosis of NSCLC patients are invasion and metastasis of tumor cells. Several studies revealed that long non-coding RNAs have a role in the regulation of cell migration and invasion. In NSCLC, high levels of SNHG6 expression were found in cells and tissues. NSCLC cell invasion is inhibited by SNHG6 knockdown. ETS1 transcription factor is upregulated by SNHG6 through its competitive binding to miR-944 and miR-181d-5p. ETS1 subsequently targets RhoA to facilitate NSCLC cell invasion by binding to WIPF1 promoter region, where it activates WIPF1 expression [33]. In NSCLC patients, SNHG6 overexpression is not just related to advanced clinical stage but to lymph node metastasis as well, making it a potential independent predictor of tumor recurrence. In A549 cells, SNHG6 promotes NSCLC cell invasive capacity by binding to miR-101-3p, thus suppressing miR-101-3p expression and promoting the expression of CDYL, its downstream target [34]. Two of these LncRNAs that inhibit tumors are MEG3 and GAS5 [35]. As MEG3 is upregulated, it promotes apoptosis via p53 and suppresses NSCLC cell migration and invasion, despite the fact that MEG3 is lowly expressed in NSCLC tissues [36]. Lv et al. said that MEG3 could be a new therapeutic target for non-small cell lung cancer (NSCLC) since it suppresses NSCLC cell motility and invasion and enhances PTEN expression through the PI3K/AKT pathway [37]. In the same way as p53-dependent and p53-independent pathways, GAS5 suppresses tumors in non-small cell lung cancers [38]. According to Dong et al., NSCLC cell proliferation, migration, and invasion are greatly affected by the downregulation of GAS5, and it can be partially reversed by the miR-205/PTEN axis. GAS5 overexpression suppresses the miR-205/PTEN axis, thus inhibiting the migration, invasion, and development of NSCLC [39]. It has been demonstrated that LNC00673 inhibits NCALD transcription and protein level via its interaction with LSD1 in NCALD promoter region, which also promotes H3K4me2 demethylation. This makes NSCLC cells more capable of migrating to other locations [40]. LSINCT5 promotes NSCLC cell motility and stabilizes the metastasis-associated transcription factor HMGA2 from proteasome-dependent degradation [41]. Besides, metastasis in lymph nodes and TNM staging are correlated with the downregulation of NKILA in NSCLC tissues. The mechanism through which NKILA transcription is regulated by the TGF-β signaling pathway is that it reduces the phosphorylation of IKBα, causing NF-κB activity restriction, and hence suppresses NSCLC cell migration and invasion [42]. Moreover, by regulating the IL-11/STAT3 signaling pathway, NKILA can suppress EMT [43].

### Apoptotic response and cell death

The regulation of apoptosis in NSCLC is greatly influenced by LncRNAs. Two main types of apoptosis pathways exist: intrinsic and extrinsic. Through the extrinsic pathway, ligands such as TNF and FasL bind to death receptors TNFR, Fas/CD95, and TRAIL, which in turn activate procaspase-8, caspase-8, and caspase-3 in a sequential fashion. The regulation of DNA fragmentation factors and caspase-activated DNases is the last step in this cascade, which leads to cell death [44]. The intrinsic process involves the selective activation of BH3 family members and the suppression of Bcl-2 proteins in response to cytotoxic stimuli such as carcinogenic stress, chemotherapeutic medications, and developmental cues. When activated, BAX and BAK, which are effectors that promote cell death, rupture the outer membrane of the mitochondria [45]. After cytochrome C binds to APAF1, which activates caspase-9, the apoptosome is formed [46]. This process is initiated by the release of ATF endonuclease and cytochrome C from the mitochondria. Protein cleavage and apoptosis execution are initiated by activated caspase-9, which in turn triggers caspase-3, caspase-6, and caspase-7. These apoptotic mechanisms are modulated by a large number of LncRNAs [47].

By enclosing the glucocorticoid receptor, a well-established tumor suppressor LncRNA known as GAS5 increases the sensitivity of cancer cells to apoptotic signals and triggers cell death. On the other hand, HOTAIR boosts anti-apoptotic gene expression, which prevents tumors from dying and makes them resistant to treatment. By controlling the miR-4677-3p/SEC61G axis and thereby reducing apoptosis, Han discovered that lnc0218 is increased in NSCLC and enhances cell proliferation. Specifically, NSCLC cells have enhanced apoptosis when lnc0218 is knocked down [48]. The researchers Wang et al. found that lncRNA-ATB increases the death of NSCLC cells by preventing the expression of miR-200a, which in turn causes the expression of β-catenin to be upregulated [49]. Apoptosis is promoted by some lncRNAs and inhibited by others. Inducing apoptosis in NSCLC cells, MEG3 binds to miR-7-5p, as demonstrated, for instance, by Yang et al. [50]. This binding process suppresses Bcl-2 expression and promotes BAX expression. A short peptide known as ATMLP is encoded by the long non-coding RNA AFAP1-AS1, according to Pei et al. This peptide promotes the malignant progression of NSCLC by localizing to mitochondria. The prognosis for a patient worsens as the serum level of ATMLP increases; this protein, and not the LncRNA itself, drives tumor growth. Methylation of m6A also controls its translation. The binding of ATMLP to NIPSNAP1 interferes with its movement into mitochondria and impacts tumor cell death, according to the mechanistic analysis [51].

## Clinical implications of LncRNAs in lung cancer

### Diagnostic biomarkers

Due in part to the fact that it is often not identified until the disease has progressed, the prognosis for lung cancer, and notably LC, is extremely dismal. Therefore, in order to aid in the early detection of lung cancer, new clinical technologies are required. Differentiating between the various forms of lung cancer is critical for directing clinical therapy choices. For diagnostic and prognostic purposes, many miRNAs have been studied [49]. Expression patterns of LncRNAs are similar to those of miRNAs with respect to tissue and developmental stage [12]. One biomarker that has shown relevance in clinical practice for predicting the amount of prostate cancer is prostate cancer gene 3 (PCA3). Prostatic secreta is a good place to look for it, which makes research on it easier [18]. Lung cancer prognosis, treatment, and diagnosis could all benefit from LncRNAs. However, studies examining the links between lncRNAs and lung cancer are still in their infancy, and LncRNAs’ predictive capabilities remain unclear. According to a recent clinical trial, MALAT1 could be a potential marker for the detection of non-small cell lung cancer [52]. With a specificity set at 96% and a sensitivity of 56% as a single biomarker for lung cancer diagnosis, MALAT1 failed to meet the criteria for an effective biomarker, according to receiver operating characteristic curves. Lung cancer prognosis is heavily dependent on histotype and clinical stage. The histotype and stage of lung cancer determine the LncRNA expression profile. High levels of a LncRNA linked to microvascular invasion in HCC (LncRNA MVIH) in non-small cell lung cancer (NSCLC) tissues are associated with advanced clinical stage and distant metastases in lung cancer, and HOTAIR is a significant, independent predictor of TNM stage [52]. It follows that LncRNAs may have potential as diagnostic tools for lung cancer in its various stages. Nevertheless, there is a present dearth of research on the use of LncRNAs as biomarkers in lung cancer. Clinical trials are necessary to establish a set of markers for various stages of lung cancer, taking into account that useful biomarkers have high specificity and enough sensitivity. Potential biomarkers for lung cancer detection include a large number of LncRNAs. Patients with lung tumors that had high levels of the long non-coding RNA Sox2ot had a worse prognosis and a shorter time to survival [53]. Further analysis revealed that lung cancer tissues had reduced expression of growth arrest-specific transcript 5 (GAS5) [54].

**Fig. 2:**
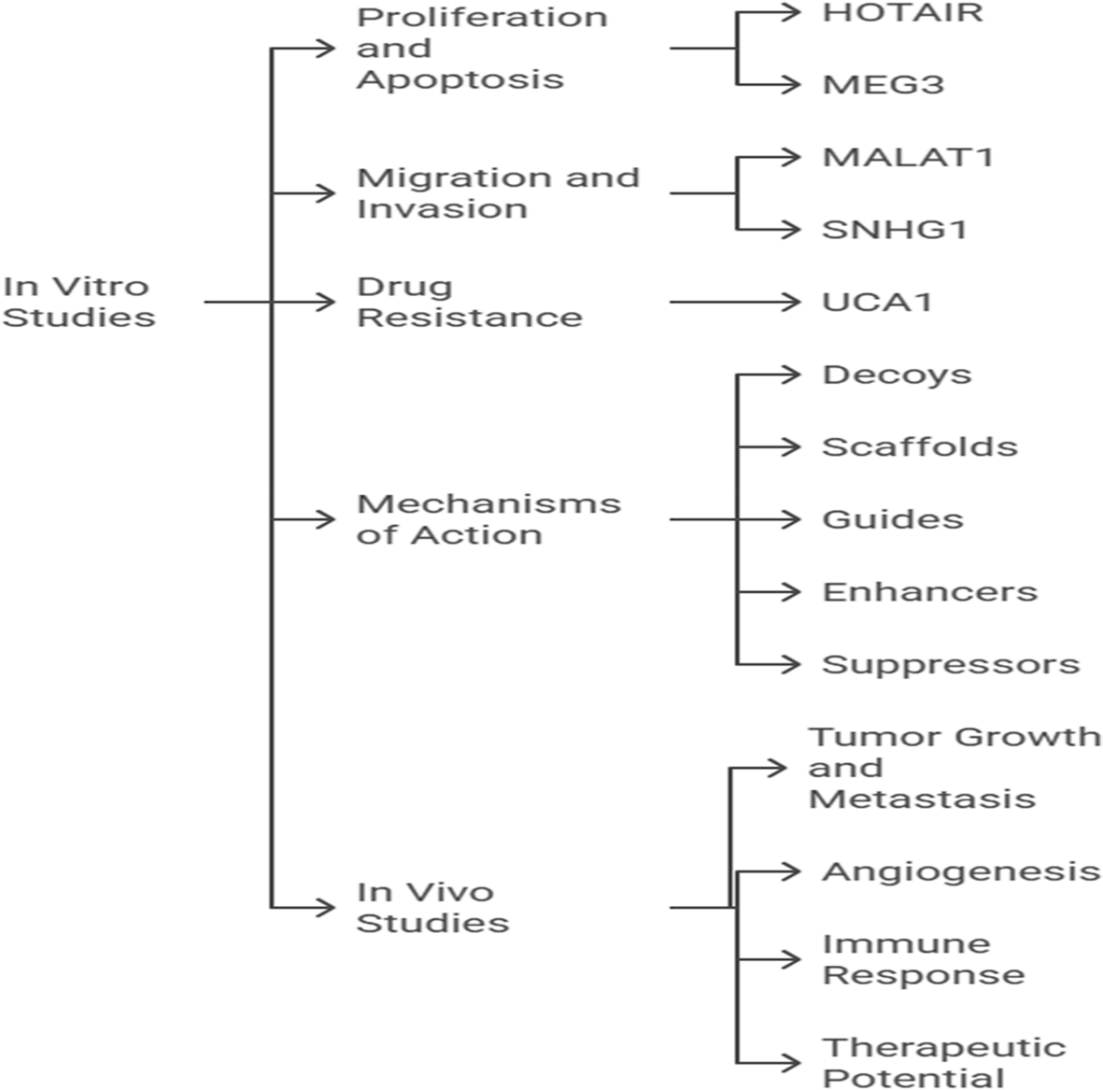
The function of LncRNAs in the development and progression of lung cancer has been determined by studies conducted in vitro and in vivo. Studies have determined that lncRNAs are able to affect a wide range of cellular processes, such as migration, invasion, angiogenesis, immune response, apoptosis, and proliferation. lncRNA targeting is a new and potential therapeutic approach for lung cancer

Another group of RNAs that do not encode proteins but that are more than 200 nucleotides in length consists of long non-coding RNAs (lncRNAs). They contribute to cell signaling, regulation of the chromatin, and gene expression, and make up a large percentage of the non-coding genome. Uncontrolled cell growth and metastasis are features of lung cancer, the most dangerous type of cancer that kills a higher number of people than any other type of cancer in the world. As shown in Fig. 2, there is mounting evidence confirming that lncRNAs are deregulated in lung cancer and involved in the initiation, development, and chemoresistance of lung cancer [54].

### Therapeutic target

There are two main types of lung cancer: SCLC and NSCLC. At this time, chemotherapy, chest radiation, and surgical removal are the mainstays in the treatment of both types of lung cancer. To improve patients’ quality of life and survival, new techniques are needed, as conventional medicines have limited efficacies. The significance of long non-coding RNAs (LncRNAs) in carcinogenesis and cancer progression is crucial, and they are highly expressed in lung cancer. Therefore, LncRNAs may likewise be promising therapeutic targets. A large body of evidence suggests that LncRNAs may have a therapeutic function [55]. Curiously, LncRNAs add to the problem of drug resistance in human lung tumor cells [26] and also promote carcinogenesis [55].

**Fig. 3:**
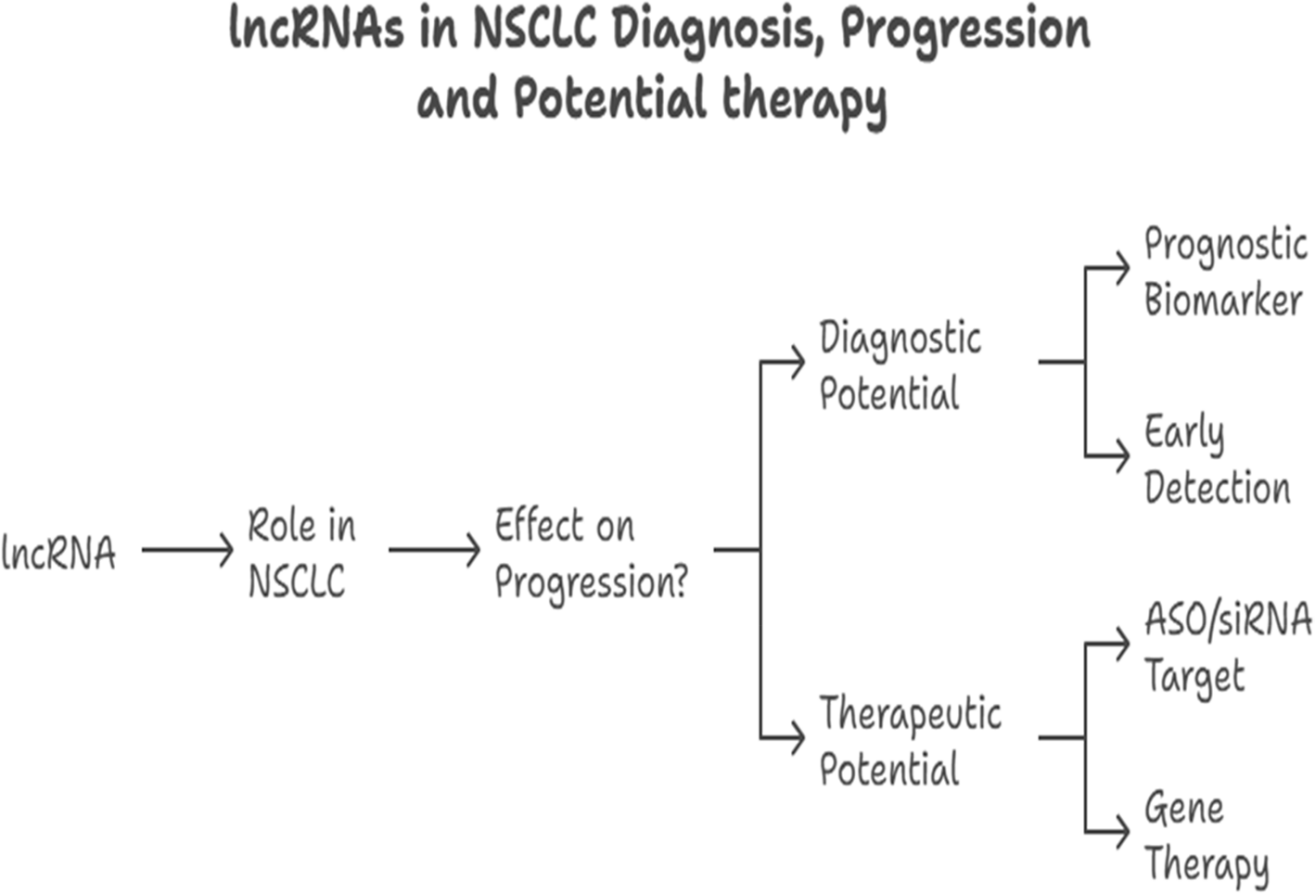
The role of the LncRNAs involved in NSCLC progression, diagnosis, and therapy would be helpful

Researchers rely on RNA interference (RNAi)-based methods the most when trying to develop effective lncRNA-based cancer therapies. One potential technique for regulating the function of certain lncRNAs is to use small interfering RNAs (siRNAs) to block these RNAs. Using siRNA to inhibit HOTAIR reduced the spread of breast cancer cells to distant sites [56]. Nevertheless, siRNAs have limited utility due to off-target effects and delivery issues [26]. The short single-stranded deoxyribonucleotide analogs known as antisense oligonucleotides (ASOs) are able to bind to target RNAs sequence-specifically through Watson–Crick base pairing. The ASO DNA/RNA complex that forms is then identified by RNase H1 and cleaved. The specificity and lack of off-target effects of ASOs are superior to those of siRNAs [56]. Injecting MALAT1-specific ASO into tumor tissue prevented lung cancer metastasis, confirming the therapeutic benefits of ASOs on LncRNAs in a recent trial [52]. Another type of ribozyme that inhibits LncRNAs is the hammerhead ribozyme. Atamamers, which are shorter oligonucleotides or peptides of DNA or RNA, influence the expression of target LncRNAs in cancer cells by binding to their secondary structure, in contrast to the aforementioned methodologies [53]. The therapy of cancer has already made great strides with the discovery of numerous medicines related to miRNA [57]. Drug safety and effective therapeutic agent delivery are two of the many current challenges to the development of novel LncRNA-based therapies, despite the fact that these agents show tremendous promise, as exhibited in Fig. 3.

## The advantage and challenge of therapeutic targeting of LncRNA

Among the novel and hopeful approaches to the treatment of NSCLC is targeted lncRNA therapy. Novel approaches to effective cancer therapies could presumably be obtained by targeted regulation of tumor-inducing lncRNAs implicated in the development and metastasis of cancer [58]. RNA-targeted treatment approaches have been a target of substantial investment during the last decades. Currently available RNA-based therapy modalities include RNA interference (RNAi), small molecule inhibitors, microRNA mimics, antisense oligonucleotides (ASOs), and CRISPR-Cas9 gene editing. ASOs and siRNAs are the most commonly applied of these [59]. Effective delivery and anti-tumor efficacy have been described using synthetic miRNA-based therapeutic agents in combination with a range of protective coating methods in preclinical models [60]. One of the initial lncRNAs to be associated with tumor metastasis was MALAT1. MALAT1 ASO subcutaneously inhibited metastatic non-small cell lung cancer significantly by lowering the number of lung nodules in a mouse model, reflecting the therapeutic potential of targeting MALAT1 in metastatic lung cancer [61]. Similarly, total survival is poorer in metastatic renal cell carcinoma patients with high lncRNA ARSR expression. Through adsorption of miR-34 and miR-449, LncARSR inhibits AXL and MET expression, further enhancing resistance to sunitinib. Intravenous administration of l ASOs against lncARSR enhanced sunitinib treatment sensitivity in an ongoing in vivo xenograft renal cell cancer model [62]. The lncRNA RAMS11 has a poor prognosis in colon cancer patients when the expression is high.

The observation that enhanced RAMS11 expression provides resistance against topoisomerase inhibitors and fluorodeoxyuridine (FUDR) was revealed in an FDA-sanctioned drug screen, which is of significant interest. Subsequently, in vitro work revealed that cellular sensitization to topoisomerase and FUDR inhibitors was increased following CRISPR-mediated reduction of RAMS11 expression [63]. In addition, long non-coding RNAs are needed for metastasis of cancer cells to specific organs. An example is the effect on osteoclastogenesis and bone resorption observed upon knockdown of LncRNA MAYA in breast cancer cells that metastasized to bones via CRISPR-mediated knockdown [64].

**Fig. 4:**
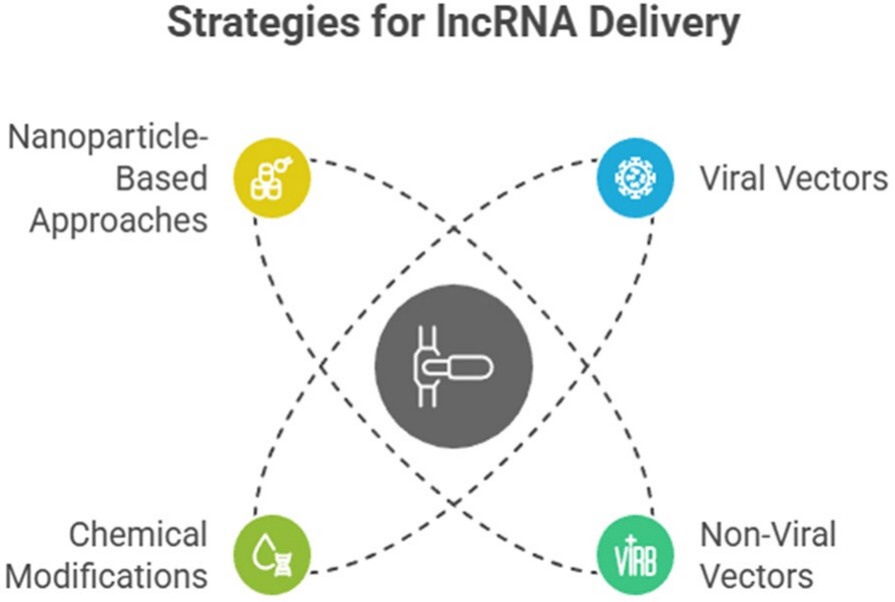
Viral vectors like adeno-associated viruses and lentiviruses deliver genetic material to cells efficiently and can be used for targeted expression. Nanoparticles are also found to work well to deliver lncRNAs by encapsulating them and delivering them to targeted cells via ligands [65]

LncRNAs have great potential as future therapeutic agents. But targeting them to the right cells or tissues is a major challenge still. In order to increase therapeutic efficacy, reduce potential toxicity, and reduce off-targeting, targeted delivery becomes absolutely critical. There are various methods of delivering lncRNAs to target locations, and each of them has positive and negative aspects. They are mainly viral vectors, non-viral vectors, and chemically modified vectors (Fig. 4) [64].

Also, studies have shown that m6A changes on LncRNAs affect carcinogenesis and treatment response, which shows that targeting ncRNAs through m6A regulatory factors could be a good way to treat cancer [66]. Additional study into other LncRNA families that could be therapeutic targets in cancer has been stimulated by the success of miRNA-based cancer research.

**Fig. 5:**
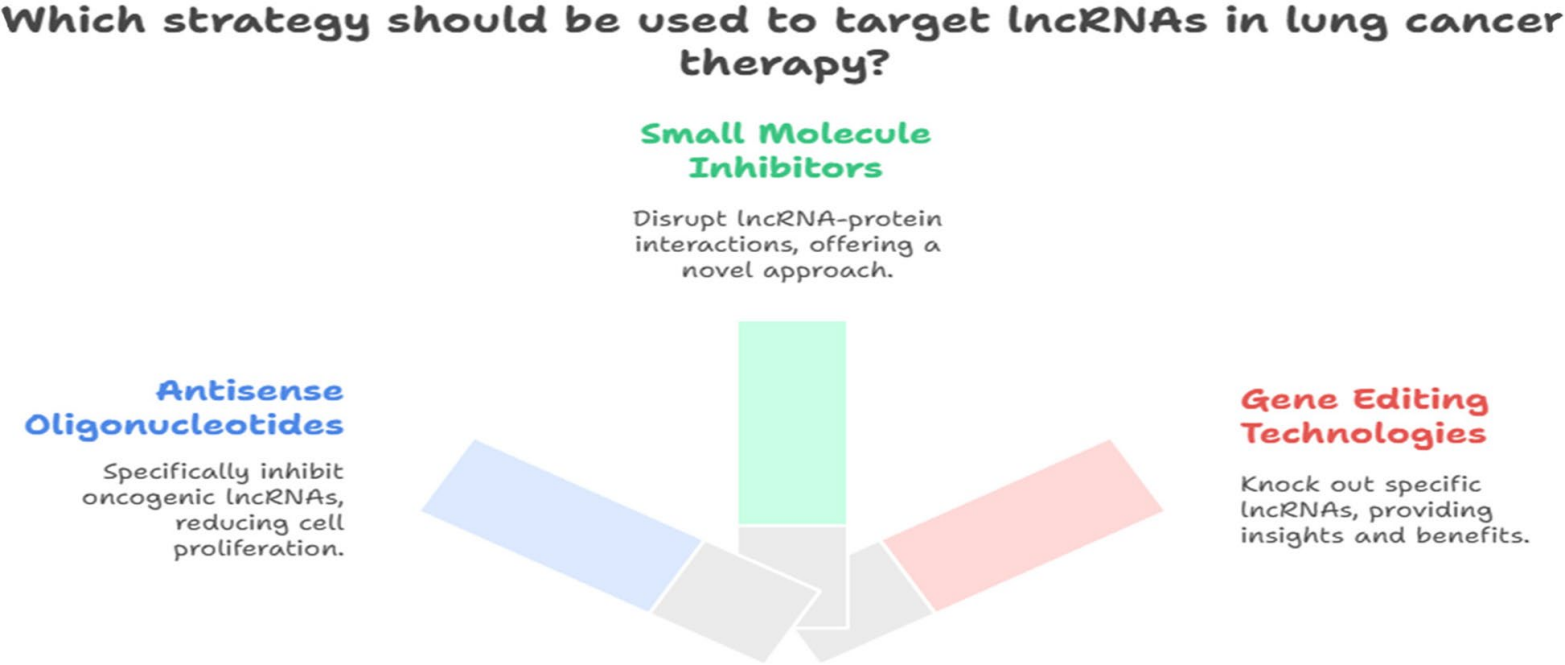
Therapeutic targeting: the biology of lung cancer is significantly influenced by long non-coding RNAs, affecting tumor behavior and patient outcomes. Their function as promising biomarkers and therapeutic targets represents an exciting area for the advancement of lung cancer diagnosis and therapy

Several obstacles stand in the way of bringing LncRNA-targeted treatments to the clinic, notwithstanding the encouraging preclinical findings. It is of the utmost importance to minimize off-target consequences when targeting LncRNAs with ASO treatments. New research suggests that ASO may lead to the premature cessation of transcription, which could harm specific RNAs. Efficacy and safety evaluations of ASO treatments should thoroughly account for this possibility [67–71]. The architectural and physiological hurdles make it extremely difficult to deliver lncRNA-targeted therapies to tumor cells in the lung. The construction of targeted therapeutics that can selectively influence a particular LncRNA without influencing others is made more complicated by the plethora of LncRNAs, which exhibit overlapping sequences and functional similarities. Although the roles of certain LncRNAs are clear, the majority are still a mystery. The development of targeted medicines is impeded, and the prediction of potential off-target effects is made more complicated by this lack of understanding. Also, these treatments should not be used without first assessing their toxicity and off-target consequences [72]. Finally, LncRNA-based therapies are still in their early stages, but their promise in cancer treatment is already shining through. In the not-too-distant future, these medicines are expected to have numerous uses as shown in Fig. 5 [73].

## How lncRNAs are involved in the regulation of treatment responses to chemotherapy, targeted therapy, and immunotherapy

LncRNAs have emerged as important regulators of chemo resistance by various mechanisms, including [74]:

- The capacity of cancer cells to carry out programmed cell death as a response to chemotherapy drugs can be influenced by LncRNAs, which regulate the apoptotic process.
- Control of drug metabolism: Some lncRNAs have the potential to influence the amount of chemotherapeutic medicines in cancer cells by influencing the expression and activity of enzymes that break down drugs.
- DNA repair mechanisms: LncRNAs can influence the potential of cancer cells to repair DNA damages caused by chemotherapy by interacting with DNA repair pathways.
- The role of long non-coding RNAs in targeted therapeutic response: Lung cancer patients harboring particular mutations, such as EGFR, ALK, or ROS1, have immensely profited from the advancement of targeted drugs, which directly act on the genetic mutations promoting cancer development.

LncRNAs are also important in mediating resistance to targeted therapy by mechanisms including [75]:

- By activating alternative signaling pathways that circumvent the targeted pathway, LncRNAs allow cancer cells to continue dividing and surviving despite the inhibition of the targeted pathway.
- Modulation of receptor tyrosine kinase (RTK) activity: Long non-coding RNAs are able to regulate RTK activity, which is the typical target of therapy drugs. This subsequently impacts their ability to signal downstream and be drug responsive.
- To fine-tune the entire responsiveness to the therapy, LncRNAs may regulate the activity or expression of downstream effectors of the targeted pathway.

LncRNAs and immune response to immunotherapy: Immunotherapy, such as immune checkpoint inhibitors (ICIs) against PD-1/PD-L1 and CTLA-4, has been an extremely promising lung cancer therapy [76].

- Immune checkpoint modulation: Certain of the long non-coding RNAs can potentially modulate immune checkpoint molecules like PD-L1, and in so doing, influence the efficiency with which ICIs modulate checkpoints and activate the immunologic response.
- LncRNAs are capable of regulating the biosynthesis of cytokines, which are signaling proteins that play important roles in immune function.
- Reversal of tumor microenvironment: Long non-coding RNAs (LncRNAs) have the potential to regulate the tumor microenvironment structure and function as well as the immune cells’ capability to invade and destroy cancer cells.

## Summary and perspective views on the future

Lung cancer LncRNAs control tumor growth, metastasis, and resistance to existing treatments, among other important biological pathways. A thorough investigation into the function of lncRNAs in signaling pathways is required, considering the interaction of coding and non-coding molecules, due to their capacity to regulate the activity of other biomolecules. A similar strategy utilizing CRISPRi and single-cell RNA-seq has lately been documented for brain cell differentiation processes [76]. Furthermore, given the growing availability of single-cell sequencing technologies, it is crucial to investigate the tumor-specific cell of origin for long non-coding RNAs (LncRNAs) such as LUCAT1, which has been found to influence immune responses. Exactly what LncRNAs do depends on their environment. So, it is crucial to conduct strong in vivo investigations to understand how LncRNAs work physiologically.

We pose targeted research questions worthy of further investigation. These questions are phrased to be actionable and address critical gaps in our current knowledge [77].

- How do different cell types within a multi-cellular tissue react to a particular stimulus or perturbation? This is what needs to be solved in the decoding phase. Targeted treatment and intervention rely on decoding cell-type-specific responses. Particular cell types must be purified and analyzed for this.
- Regulatory network clarification: Which regulatory networks are most centrally involved in modulating cellular behavior, and how do those networks adapt to respond to disease and environmental signals? New therapeutic targets and mechanisms for modulating cellular behavior can be uncovered by mapping these networks.
- Examining cell–cell communication: How do cells in an organ or tissue talk to each other, and how does that talk affect the stability of the organ or tissue and the course of disease? Understanding cell–cell communication is necessary to come up with treatments targeted against intercellular signaling pathways.
- What do long non-coding RNAs (e.g., microRNAs) and other non-coding RNAs (e.g., snRNAs) do with respect to regulating gene expression and cellular function? Misregulation of non-coding RNAs has been associated with an unprecedented list of disorders, and their role as regulators of cellular activity is being recognized more and more.
- An exploration of how epigenetic changes function: What is the effect of changes in epigenetics (for example, histone modification and DNA methylation) on gene expression and cellular phenotype? The environment affects epigenetic changes, which play a critical role in gene regulation.
- How cellular processes shift over time in response to environmental inputs or due to alterations in development? Design of drugs targeting particular stages of disease pathology requires understanding the dynamics of cellular processes.
- Identification of new biomarkers: Is it possible to identify new biomarkers, which can be utilized for disease diagnosis, prediction of therapy response, or monitoring of disease progression? For developing improved treatments and providing personalized medicine, biomarkers play a vital role.

Finally, whereas lncRNAs have been shown to have a role in therapeutic resistance, their potential involvement in the persister cell state, also known as minimal residual illness, has not been investigated. Important survival mechanisms driving treatment failure and illness recurrence could be illuminated by such studies. Because of how stable they are in circulation, LncRNAs have great potential as disease biomarkers. They may be useful for early illness detection and temporal circular lncRNA detection to track treatment efficacy, both of which warrant further investigation. Routine monitoring in groups of patients with advanced cancer could benefit from such an approach [78–84].

## Data Availability

No datasets were generated or analysed during the current study.

## Abbreviations

lncRNA: Long non-coding RNA
Mir RNA: MicroRNA
LC: Lung cancer
NSCLC: Non-small cell lung cancer
